# Longitudinal alterations of gut mycobiota during 2 years after COVID-19 and its correlation with pulmonary sequela

**DOI:** 10.1128/spectrum.03007-24

**Published:** 2025-05-23

**Authors:** Qianhan Xie, Jiali Ni, Ling Yu, Wanru Guo, Cheng Ding, Fengjiao Wang, Yechen Wu, Kaijin Xu, Yanfei Chen

**Affiliations:** 1 State Key Laboratory for Diagnosis and Treatment of Infectious Diseases, National Clinical Research Center for Infectious Diseases, Collaborative Innovation Center for Diagnosis and Treatment of Infectious Diseases, The First Affiliated Hospital, College of Medicine, Zhejiang University 12377, Hangzhou, Zhejiang, China; 2 Jinan Microecological Biomedicine Shandong Laboratory 661980 , Jinan, Shandong, China; Nanchang University, Nanchang, Jiangxi, China

**Keywords:** post-acute COVID-19 syndromes, pulmonary sequelae, gut mycobiota, convalescence, biomarker

## Abstract

Pulmonary sequelae associated with COVID-19 are an increasing concern. Emerging evidence suggests that gut dysbiosis may contribute to post-acute COVID-19 syndromes; yet, the specific link between gut mycobiota and pulmonary sequelae remains unclear. We conducted a prospective study with 46 COVID-19 patients and 37 healthy controls. Longitudinal assessments of gut mycobiota were performed at admission, 6 months, 1 year, and 2 years post-discharge using internal transcribed spacer (ITS) 3–4 sequencing. At the 2-year follow-up, pulmonary function tests and chest computed tomography (CT) scans were also performed and correlated with gut mycobiota. While pulmonary function remained well-preserved, radiographic abnormalities persisted in a small subset of patients at 2 years. During the acute phase, fungal richness, as indicated by Chao1 and Shannon indices, was significantly lower than in controls, but gradually returned to normal levels after 6 months of recovery. Genus *Candida*, elevated during the acute phase, reverted to control levels after 6 months. At the 2-year follow-up, there was an increased presence of *Hanseniaspora*, *Issatchenkia*, and *Saturnispora* and a decreased presence of *Aspergillus*, *Penicillium*, and *Rhodotorula*. In addition, *Pichia*, *Saccharomycopsis*, and *Cladosporium* were especially enriched in patients without residual CT abnormalities at 2 years. At 2 years post-discharge, significant correlations were observed between pulmonary function and gut mycobiota, including negative correlations between *Hanseniaspora* and PEF, as well as negative correlations between *Saturnispora* and DLCO. This study reveals the longitudinal shifts in gut mycobiota over 2 years post-discharge in COVID-19 patients. Significant correlations were observed between pulmonary function and the gut mycobiota, suggesting a potential detrimental effect on lung function, offering potential insights into the pathogenesis of post-acute COVID-19 syndromes.

**IMPORTANCE:**

This article elucidates the intricate process of gut mycobiota reconstitution within a 2-year timeframe following COVID-19 infection and establishes a significant link between the gut mycobiota and the recuperation of pulmonary function. Our research suggests that the gut mycobiota could be utilized as diagnostic indicators for post-acute COVID-19 syndromes and may offer avenues for developing therapeutic interventions.

## INTRODUCTION

The COVID-19 pandemic has had profound and far-reaching impacts on global health (1). Various SARS-CoV-2 variants, including Alpha, Delta, and Omicron, have contributed to successive waves of infections. As these newer strains exhibit reduced virulence, attention has shifted toward understanding the recovery process and the development of post-acute COVID-19 syndromes (PACS). Mounting evidence suggests that many individuals recovering from COVID-19 experience long-term complications or persistent symptoms (2, 3). However, the factors and mechanisms underlying PACS remain poorly understood.

The lungs are the primary organs affected by SARS-CoV-2 infection, and long-term pulmonary sequelae are a significant concern. COVID-19 has been linked to a range of persistent respiratory complications, from ongoing symptoms and radiographic abnormalities to impaired respiratory function, vascular issues, and pulmonary fibrosis (4–7). Increasing evidence indicates that physiological and radiographic abnormalities persist in some COVID-19 patients even 12 months after discharge (5, 8). Our previous study found that around half of the patients exhibited residual pulmonary CT abnormalities a year after discharge, with ground-glass opacity and reticular patterns as the predominant radiologic findings (9). Factors associated with poorer pulmonary recovery include female sex, pre-existing lung disease, smoking history, and severe disease presentation (8).

Although the lungs are the most affected organ, SARS-CoV-2 is capable of infecting cells all over the body expressing ACE2, including enterocytes in the gut, which is relevant to dysbiosis (10). Persistent gut dysbiosis may play a role in post-COVID conditions. Long-term alterations in gut microbiota have been associated with ongoing gastrointestinal and neurological symptoms, suggesting that the gut-lung axis—an interactive pathway linking the gut and respiratory system—may be disrupted (11). Dysbiosis in the gut can exacerbate respiratory symptoms and vice versa, highlighting the potential of microbiota restoration as a therapeutic strategy to alleviate long-COVID symptoms (12). Gut dysbiosis exacerbates COVID-19 through multiple mechanisms, including microbial translocation, systemic inflammation, immune dysregulation, and metabolic disturbances (13). The intestinal microbiota is a complex community of bacteria, archaea, viruses, protists, and fungi, with emerging evidence underscoring the importance of fungi as a key component (14). Studies indicate that the gut mycobiota (the fungal portion of the microbiota) may influence COVID-19 outcomes and recovery. For example, patients with severe COVID-19 have shown reduced fungal diversity and an increased presence of *Candida* species compared to healthy individuals, who typically exhibit a more balanced fungal community dominated by non-pathogenic fungi like *Aspergillus* (15, 16). However, little was known about the recovery process of gut mycobiota and its relationship with post-COVID complications.

Given the close correlation between gut mycobiota and pulmonary sequelae, along with limited treatment options for PACS, understanding microbial changes during recovery may aid in early identification and intervention for affected patients (17). In this study, a cohort of COVID-19 patients discharged in April 2020 was prospectively followed for 24 months. The objectives were to (i) characterize longitudinal changes in gut mycobiota over 2 years post-discharge in COVID-19 patients and (ii) examine associations between gut mycobiota and pulmonary sequelae.

## MATERIALS AND METHODS

### Study design and participants

This was a prospective, longitudinal follow-up study involving 34 COVID-19 patients who were discharged from the First Affiliated Hospital, Zhejiang University School of Medicine, Hangzhou, China, between February 1 and March 15, 2020. These patients were followed up at 6 months, 1 year, and 2 years post-discharge.

A control cohort of 36 healthy subjects served as the uninfected controls, matched for age, gender distribution, BMI, and the prevalence of comorbidities with the COVID-19 cohort. Exclusion criteria included a history of inflammatory bowel disease, gastrointestinal surgery, or the use of antibiotics, probiotics, or prebiotics within 1 month prior to the study’s start.

COVID-19 patients were classified by disease severity according to the Chinese Clinical Guidance for COVID-19 Pneumonia Diagnosis and Treatment (7th edition) (18). Briefly, patients were grouped into four severity levels: mild, moderate, severe, and critical illness. Mild illness was defined by mild clinical symptoms without radiological signs of pneumonia, while ordinary illness included symptoms of fever, respiratory issues, and radiological evidence of pneumonia. Severe illness was indicated by at least one of the following: respiratory rate ≥30 bpm, arterial oxygen saturation (SaO2) ≤93% at rest, partial pressure of oxygen (PaO2)/FiO2 ≤300 mmHg, or more than 50% lesion progression on chest radiograph within 24–48 hours. Critical illness was defined by the presence of respiratory failure requiring mechanical ventilation, shock, or multi-organ failure requiring intensive care. For analysis, mild and ordinary cases were grouped as the mild group, while severe and critical cases were combined into the severe group.

### DNA extraction and ITS sequencing

During hospitalization or follow-up visits, patients provide stool samples. The samples, about the size of a soybean, are placed in sterile collection tubes, kept in ice boxes at 4°C, and transferred to the laboratory within 1 hour. There, they are portioned and stored at −80°C for later analysis as per research requirements. To minimize the risk of infection from live viruses in stool samples and prioritize biosafety level 3 laboratory resources for clinical use, all experimental procedures were conducted in a biosafety level 2 laboratory. Fecal samples from patients in the acute phase were first heated at 56°C for 30 minutes to inactivate any viruses present. Samples from the recovery phase and controls were processed under the same conditions to ensure methodological consistency. Microbial DNA was extracted from these samples using the PowerSoil Pro Kit (Qiagen, California, USA) according to the manufacturer’s protocol. Fungal internal transcribed spacer (ITS) regions (ITS 3–4) were amplified for sequencing using the ITS3F and ITS4R primers. The PCR products were purified by electrophoresis on a 2.0% agarose gel with the AxyPrep DNA Gel Extraction Kit (Axygen Biosciences, Union City, CA, USA) and quantified using a Quantus Fluorometer (Promega, USA) according to the manufacturer’s instructions. Purified amplicons were then subjected to paired-end sequencing (2 × 300) on the Illumina MiSeq PE300 platform (Illumina, San Diego, California, USA). Detailed protocols for fungi genomic DNA extraction, PCR amplification, Illumina MiSeq sequencing, and bioinformatic analysis were followed as described previously (19). Briefly, original ITS gene sequencing reads were analyzed and quality-filtered with Trimmomatic, then merged using FLASH, following these criteria: (i) 300 bp reads were truncated at any site receiving an average quality score <20 over a 50 bp sliding window. Truncated reads shorter than 50 bp were discarded, as were those with ambiguous characters. (ii) Overlapping sequences were assembled only if their overlapping length exceeded 10 bp, with a maximum mismatch ratio of 0.2 in the overlapping region. Unassembled reads were discarded. OTU clustering was performed at a 97% similarity threshold using UPARSE 7.1 (http://drive5.com/uparse). Chimeric sequences were identified and removed. The RDP Classifier (http://rdp.cme.msu.edu/) analyzed each OTU’s representative sequence against the UNITE database for taxonomic classification. To minimize the effects of sequencing depth on alpha and beta diversity measures, the number of ITS gene sequences from the different subjects was equalized by random subtraction to 39,495 (equal to the subject with the smallest sample size). The sequence data processing, alpha diversity, and beta diversity were done on the Majorbio Cloud Platform (www.majorbio.com).

### Radiological imaging

High-resolution CT (HRCT) scans were performed to assess residual radiological abnormalities. All radiographic examinations were conducted using standardized techniques on the same CT equipment. The CT images were independently reviewed by two radiologists, each with over 5 years of experience, following previously described protocols (20).

### Pulmonary function tests

Two years after hospital discharge, pulmonary function parameters, including forced vital capacity (FVC), forced expiratory volume in the first second (FEV1), peak expiratory flow (PEF), FEV1/FVC ratio, diffusing capacity for carbon monoxide (DLCO), diffusing capacity per unit alveolar volume (DLCO/VA), total lung capacity (TLC), residual volume (RV), and the RV/TLC ratio, were measured using the SensorMedics Vmax System (USA). Results were expressed as percentages of predicted normal values (21). Pulmonary function tests (PFTs) were not conducted for patients in the acute phase during hospitalization, as they were too weak, and to minimize potential cross-infection risks.

### Statistical analyses

Statistical analyses were carried out with SAS version 9.4 (SAS Institute Inc., Cary, NC, USA). For inter-group comparisons between each time point and the healthy control group, quantitative data were analyzed using the unpaired two-sample Student’s t-test or Mann-Whitney U test, while category data were assessed with Fisher’s exact test. For intra-group comparisons of the same sample across different time points, a paired Student’s t-test or a Mann-Whitney U test was applied for quantitative data, and the McNemar test was used for categorical data. The Spearman correlation coefficients between the top 10 abundant fungal genera and the pulmonary function index were computed and visualized on a heat map. The significance levels of these coefficients were adjusted using Bonferroni’s correction. The heat map uses different colors to indicate the values of R, with the color range for different R values shown on the right side of the legend. Values of *P* < 0.05 are marked with an asterisk (“*”).

## RESULTS

### Clinical characteristics of patients and recovery of pulmonary function

Between February and March 2020, we recruited 34 patients from the First Affiliated Hospital of Zhejiang University School of Medicine in Hangzhou, China, and followed them for 2 years. The median age of the patients was 49 years (IQR 37.25–58.5 years), and 24 (70.6%) were male. Hypertension (29.4%) was the most common comorbidity, followed by type 2 diabetes mellitus (9.7%). During hospitalization, most patients (67.6%) experienced severe or critical illness (Table 1).

**TABLE 1:** Clinical characteristics of patients in study^*a*^

Characteristic	COVID-19 patients (*n* = 34)	Healthy controls (*n* = 36)	*P*-value
Age, years	49 (37.25, 58.5)	50 (34.75, 55.25)	0.835
Male, *n* (%)	24 (70.6%)	24 (66.7%)	0.724
BMI, kg/m^2^	23.8 (21.4, 24.9)	22.9 (21.0, 24.3)	0.721
Smoking history	4 (9.76%)	5 (13.9%)	0.791
Comorbidities			
Hypertension, *n* (%)	10 (29.4%)	10 (27.8%)	0.879
Type 2 diabetes, *n* (%)	4 (9.7%)	5 (13.9%)	0.791
Coronary artery heart disease, *n* (%)	2 (4.9%)	3 (8.3%)	0.691
Illness progress			
Severe illness during hospitalization	23 (67.6%)		
Duration from illness onset to hospital admission, days	7 (5, 9)		
Nasopharyngeal viral RNA shedding duration, days	15 (13, 19)		
Duration of hospitalization, days	17 (14, 23)		
ICU admission, *n* (%)	3 (8.8%)		
HFNC, *n* (%)	6 (17.6%)		
Corticosteroid treatment, *n* (%)	21 (61.8%)		

^*a*^ The quantitative data are shown as median data and interquartile range data in brackets. The occurrence data are shown as no. (%). Values indicate no. of positive results/total no. of patients with available assay results. Student’s t-test or Mann-Whitney U test was used for the quantitative data when applicable. Fisher’s exact test was used for category data. Abbreviations: BMI, body mass index; ICU, intensive care unit; HFNC, high flow nasal catheter oxygen therapy.

Pulmonary CT scans were performed on 15 patients 2 years after discharge. These patients were divided into two groups based on the presence (*n* = 7) or absence (*n* = 8) of residual lesions on CT 1 year after discharge (Table 2). At the 2-year follow-up, patients with residual findings on lung CT had a significantly longer interval from onset to hospital admission compared to those without residual findings (median of 3.5 days in the non-residual group vs. 6 days in the residual group, *P* = 0.037). Patients with residual CT findings also showed a slightly higher erythrocyte sedimentation rate (ESR) during the acute phase than those without residual findings, though the difference was not statistically significant (*P* = 0.097). While some patients had residual findings on lung CT, pulmonary function tests revealed no significant differences between the groups. However, patients with CT residuals had a slightly lower residual volume than those without, indicating a potential delay in lung elasticity recovery (*P* = 0.077).

**TABLE 2:** Comparison of clinical characteristics between subjects with or without residual CT abnormalities^*a*^

Characteristic	No residual lesions (*n* = 8)	Presence of residual lesions (*n* = 7)	*P-*value^*c*^
Age, years	45 (34.5, 53.75)	48 (39.5, 56.5)	0.232
Male, *n* (%)	5 (62.5%)	4 (57.1%)	0.832
BMI, kg/m^2^	23.5 (21.8, 26.2)	24.2 (23.7, 25.8)	0.798
Drinking history, *n* (%)	0 (0.0%)	0 (0.0%)	1.000
Current smoker, *n* (%)	0 (0.0%)	1 (14.3%)	0.268
Hypertension, *n* (%)	2 (25.0%)	3 (42.9%)	0.464
Type 2 diabetes, *n* (%)	0 (0.0%)	0 (0.0%)	1.000
Duration from illness onset to hospital admission, days	3.5 (1, 5.5)	6 (5, 7)	**0.037**
Severe illness during hospitalization	1 (12.5%)	2 (28.6%)	0.437
Nasopharyngeal viral RNA shedding duration, days	13 (11, 18.25)	19 (16.5, 20)	0.283
Peak CT score during hospitalization	6 (2, 9.5)	8.5 (6.75, 11.75)	0.665
Corticosteroid treatment, *n* (%)	5 (62.5%)	6 (85.7%)	0.310
Secondary bacterial infection, *n* (%)	0 (0.0%)	1 (14.3%)	0.268
ICU admission, *n* (%)	0 (0.0%)	0 (0.0%)	1.000
HFNC, *n* (%)	1 (12.5%)	0 (0.0%)	0.333
Laboratory indicators of the acute phase			
Lymphocyte count (×10^9^/L)	0.95 (0.75, 1.2)	1 (0.65, 2.05)	0.768
D-dimer, mg/L	213.5 (170, 353.5)	326 (222.5, 402.5)	0.463
ALT, U/L	39 (21, 52.25)	19 (16, 29.35)	0.124
AST, U/L	21 (19.5, 41.75)	18 (13.05, 25)	0.150
CRP, mg/L	6.865 (3.5475, 11.5275)	20.94 (9.435, 44.65)	0.218
IL-6 (pg/mL)	19.33 (5.515, 51.295)	13.22 (5.2, 28.455)	0.462
ESR, mm/h	13 (7.25, 21.75)	27 (10, 76)	0.097
SaO2	0.9925 (0.98025, 0.996)	0.979 (0.96, 0.98)	0.175
PFTs at 2-year follow-up*^b^*			
FVC %	98.5 (93.75, 109)	95 (88, 100)	0.955
FEV1 %	97.5 (90, 105)	102 (90, 106)	0.356
PEF	99.5 (76.5, 102.25)	88 (76.5, 99.5)	0.877
FEV1/FVC	79.85 (76.35, 84.475)	83.6 (81.75, 84.65)	0.402
DLCO %	113.5 (110.75, 128.5)	106 (98, 122.5)	0.537
TLC %	107 (98.25, 119.5)	95 (89.5, 101.5)	0.359
RV %	140 (130, 146.75)	106 (101.5, 113.5)	0.077

^*a*^ The quantitative data are shown as median data and interquartile range data in brackets. The occurrence data are shown as no. (%). Values indicate no. of positive results/total no. of patients with available assay results. Student’s t-test or Mann-Whitney U test was used for the quantitative data when applicable. Fisher’s exact test was used for category data. Abbreviations: BMI, body mass index; COPD, chronic obstructive pulmonary disease; ICU, intensive care unit; ALT, alanine aminotransferase. AST, aspartate aminotransferase; ESR, erythrocyte sedimentation rate; CRP, C-reactive protein; IL-6, interleukin-6; SaO2, oxygen saturation; HFNC, high flow nasal catheter oxygen therapy; PFTs, pulmonary function tests; FVC, forced vital capacity; FEV1, forced expiratory volume in the first 1 second of expiration; PEF, peak expiratory flow; DLCO, diffusing capacity of the lung for carbon monoxide; TLC, total lung capacity; RV, residual volume.

^*b*^ Pulmonary function tests were expressed as a percentage of the predicted value.

^*c*^ P value was analyzed using the unpaired two-sample Student’s T-Test or Mann-Whitney U test, while category data were assessed with Fisher's exact test. Values of *P* < 0.05 are marked with bold.

Eighteen patients underwent pulmonary function tests 2 years after discharge, with overall lung function well-preserved. Only one patient (5.5%) had a total lung capacity (TLC) below 80%, and one patient (5.5%) showed a pulmonary diffusion abnormality (DLCO < 80% predicted), a marked decrease from 6 months post-discharge (10 patients, 30.3%, *P* = 0.023). Significant improvement in DLCO was observed at 2 years (median 95, IQR 89.5–109.75) compared to 6 months post-discharge (median 88, IQR 77–95, *P* < 0.001). No significant differences were noted in other pulmonary function test (PFT) parameters at 6- and 12-month follow-ups (Table S1).

### Overall composition of gut mycobiota of patients recovering from COVID-19

**Fig 1:**
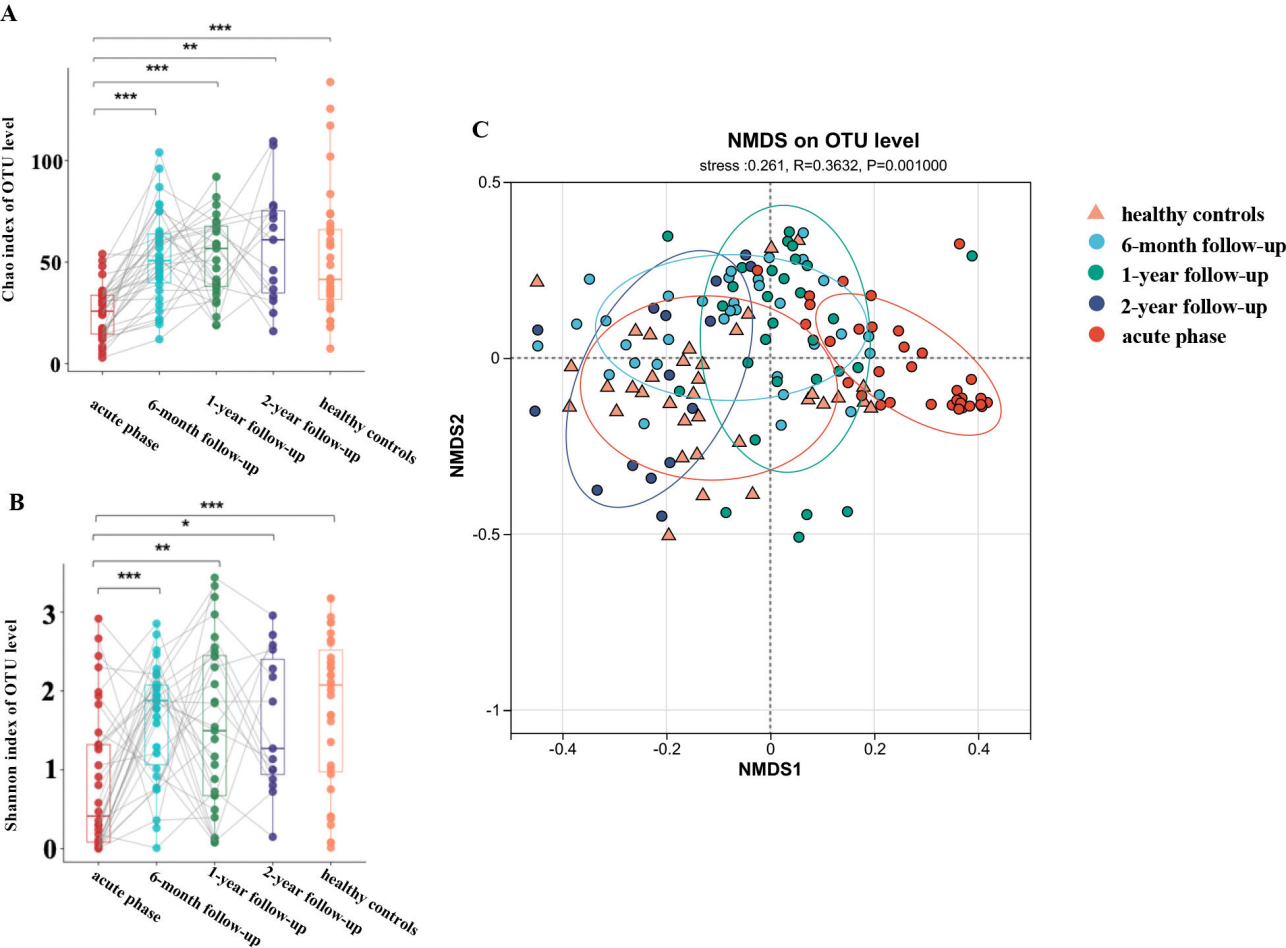
Longitudinal profiling of fecal mycobiota in patients with COVID-19. (A-B) α-diversity of the Chao 1 index, which indicates microbial richness, was markedly reduced in these patients in the acute and recovery phase vs. healthy controls (*P* < 0.001). For the same sample at different time points, a paired Student’s t-test or Mann-Whitney U test was used depending on whether the data were normally distributed. For comparing each time point with the healthy control group, an unmatched two-sample Student’s t-test or Mann-Whitney U test was used when applicable. (C) Non-metric multidimensional scaling (NMDS) demonstrated significant differences between the acute phase (red dots), 6-month follow-up (blue dots), 1-year follow-up (green dots), 2-year follow-up (deep blue dots), and healthy controls (yellow triangles) based on the Bray-Curtis distance of OTUs (stress = 0.261, ANOSIM R = 0.0.3632, *P* = 0.001).

Among the 34 patients with COVID-19, a total of 113 stool samples were collected for fecal mycobiota analysis at admission (*n* = 34), 6-month follow-up (*n* = 34), 1-year follow-up (*n* = 30), and 2-year follow-up (*n* = 15). Samples from healthy controls were only collected at a single time point. Using samples from the acute phase and subsequent follow-ups, we traced the recovery process of gut microbiota and mycobiota. Fungal richness, as measured by the Chao1 index, was significantly lower during the acute phase compared to controls (25.4 ± 13.2 vs. 52.3 ± 31.3, *P* < 0.001), along with a decrease in the Shannon diversity index (0.84 ± 0.89 vs. 1.76 ± 0.98, *P* < 0.001). However, both fungal richness and diversity gradually returned to levels comparable to healthy controls after 6 months of convalescence (Fig. 1A and B). Furthermore, we found no significant difference in diversity among the mild group during the acute, recovery phases, and healthy controls. However, in the severe group, alpha diversity significantly decreased during the acute phase but recovered by the 6-month mark. These results indicate that disease severity impacts the fungal microbiota (Fig. S1).

Consistent with the alpha diversity measures, non-metric multidimensional scaling (NMDS) based on Bray–Curtis dissimilarities revealed significant differences in mycobiota community composition during the acute phase compared to controls. Over the 2-year follow-up, the community composition gradually approached that of the controls (Fig. 1C). The mycobiota community compositions at the 6-month, 1-year, and 2-year follow-ups exhibited nearly identical cluster centroids, which were distinct from those observed during the acute phase, indicating a slow recovery rate after 6 months (Fig. 1C**,** ANOSIM, R = 0.0.3632, *P* = 0.001). Compared to the gut mycobiota during the convalescence and healthy controls, the samples collected during the acute phase showed a more similar structure, characterized by a smaller beta-diversity distance, suggesting that individual differences in the gut mycobiome during the acute phase were reduced (Fig. S2).

### Longitudinal analysis of mycobiota changes during recovery

**Fig 2:**
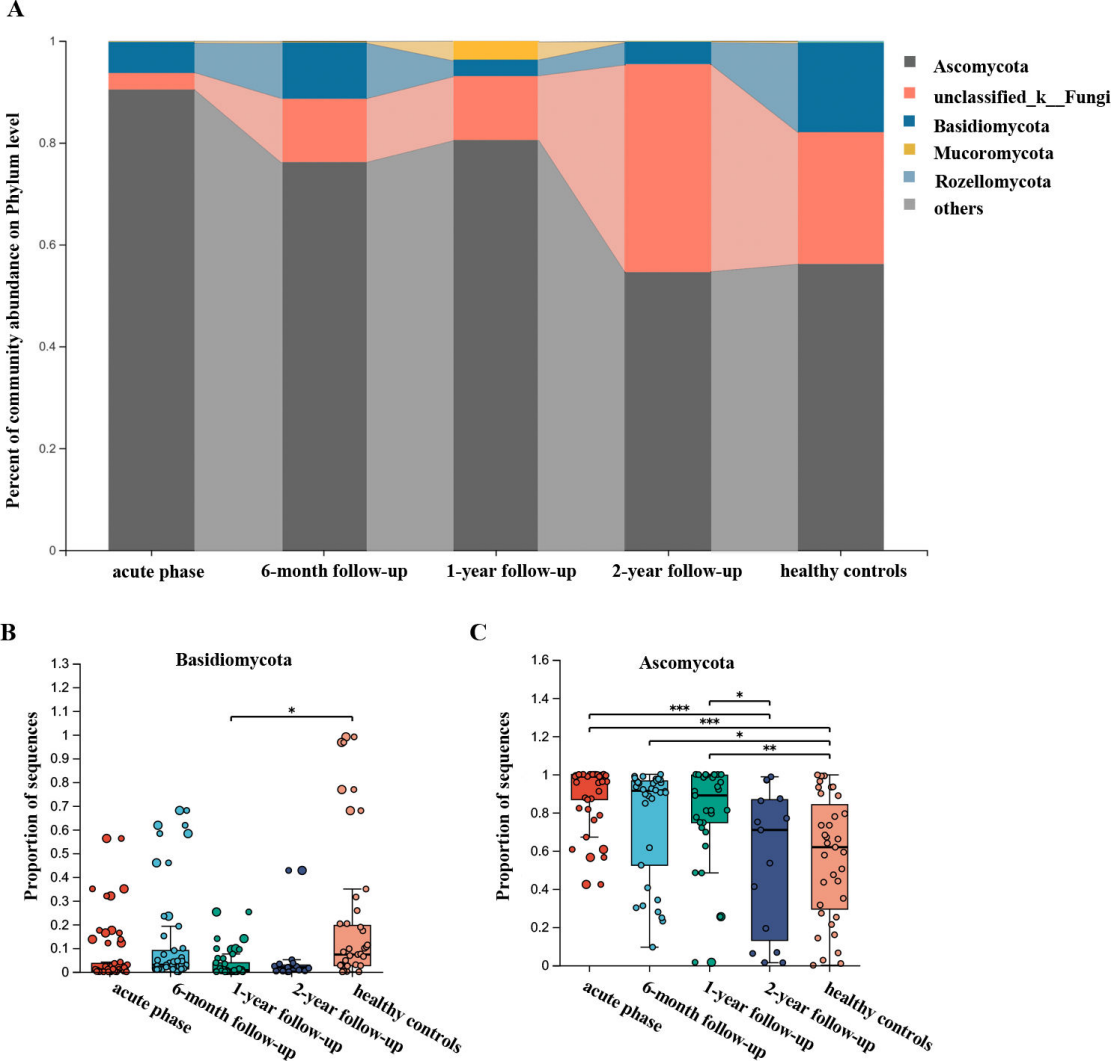
Longitudinal analysis of the gut mycobiota at the phylum level in patients with COVID-19. (**A**) Average relative abundance of the top five phyla. The shaded part connects the abundance change of the same fungal taxa between two adjacent time points to better illustrate the dynamic trend. (**B and C**) Comparative analysis of fungi phyla with significant differences across different timepoints. For the same sample at different time points, a paired Student’s t-test or Mann-Whitney U test was used depending on whether the data were normally distributed. For comparing each time point with the healthy control group, an unmatched two-sample Student’s t-test or Mann-Whitney U test was used when applicable.

**Fig 3:**
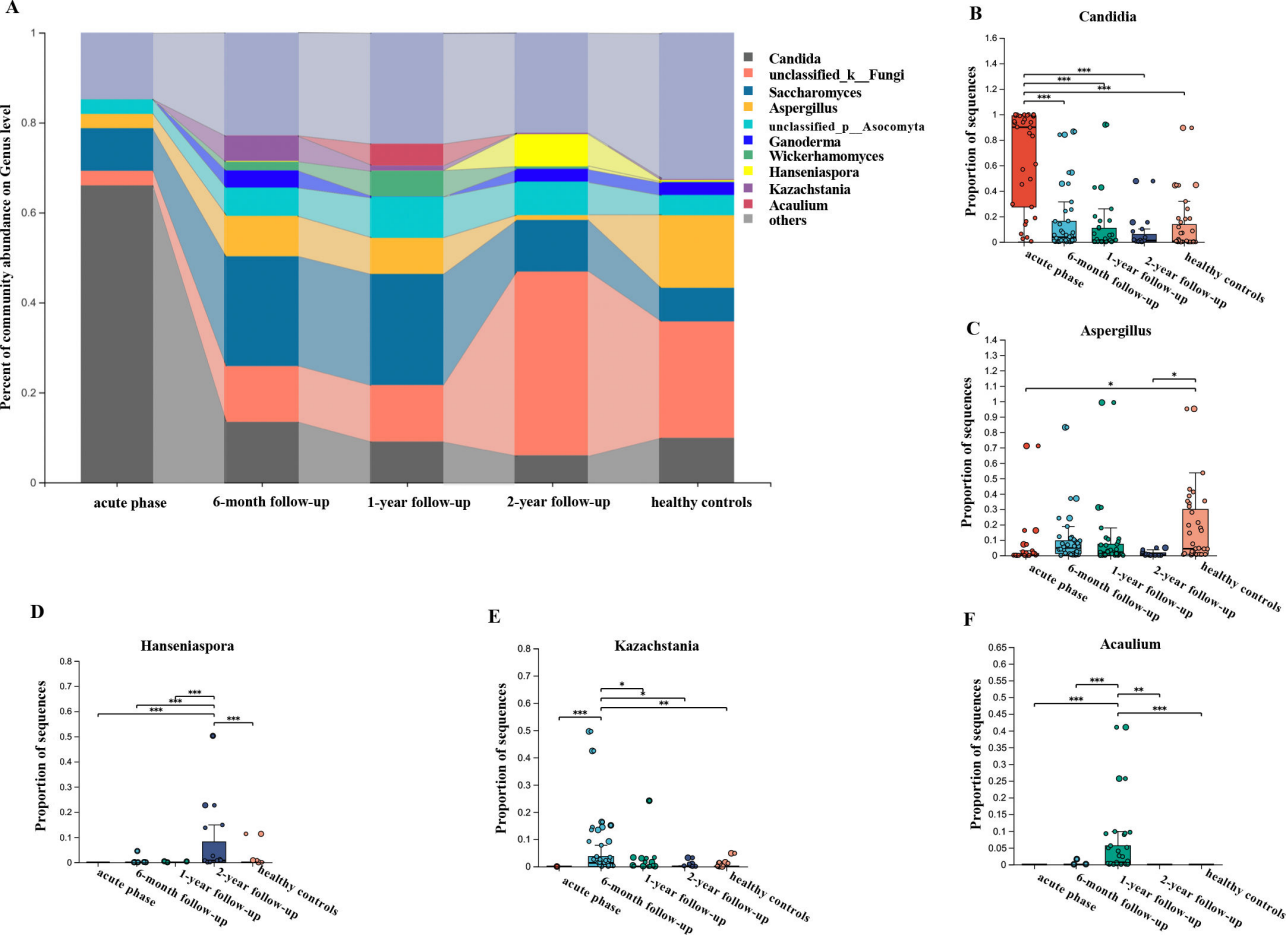
Longitudinal analysis of the gut mycobiota at the genus level in patients with COVID-19. (**A**) Average relative abundance of the top 10 most prevalent genera. The shaded part connects the abundance change of the same fungal taxa between two adjacent time points to better illustrate the dynamic trend. (**B–F**) Comparative analysis of fungal genera with significant differences across different timepoints. For the same sample at different time points, a paired Student’s t-test or Mann-Whitney U test was used depending on whether the data were normally distributed. For comparing each time point with the healthy control group, an unmatched two-sample Student’s t-test or Mann-Whitney U test was used when applicable.

We observed dysbiosis and heterogeneity in the fecal mycobiota profiles defined by the top five phyla (Fig. 2) and the ten most prevalent genera (Fig. 3) across longitudinal sampling time points. The relative abundance of representative taxa was assessed to evaluate changes during recovery. *Ascomycota* significantly increased during the acute phase but decreased over the course of 1 year, without returning to control levels. By the 2-year follow-up, however, *Ascomycota* had returned to normal control levels. The abundance of unclassified k-fungi was significantly lower during the acute phase compared to the control group, and it did not return to normal levels until the 2-year mark.

At the genus level, *Candida*, which was overrepresented during the acute phase, returned to levels seen in controls after 6 months of convalescence. By contrast, *Aspergillus*, which was depleted during the acute phase, exhibited a non-significant increase from the acute phase to the 2-year recovery period (Fig. 3B and C). Notably, *Kazachstania* and *Acaulium* were enriched at the 6-month and 12-month follow-ups, respectively (Fig. 3E and F). These results suggest that following the disruption caused by COVID-19, the fungal community may undergo distinct changes and may not fully revert to the pre-infection state.

### Residual taxon alterations of gut mycobiota at 2-year follow-up and the associations between pulmonary index

**Fig 4:**
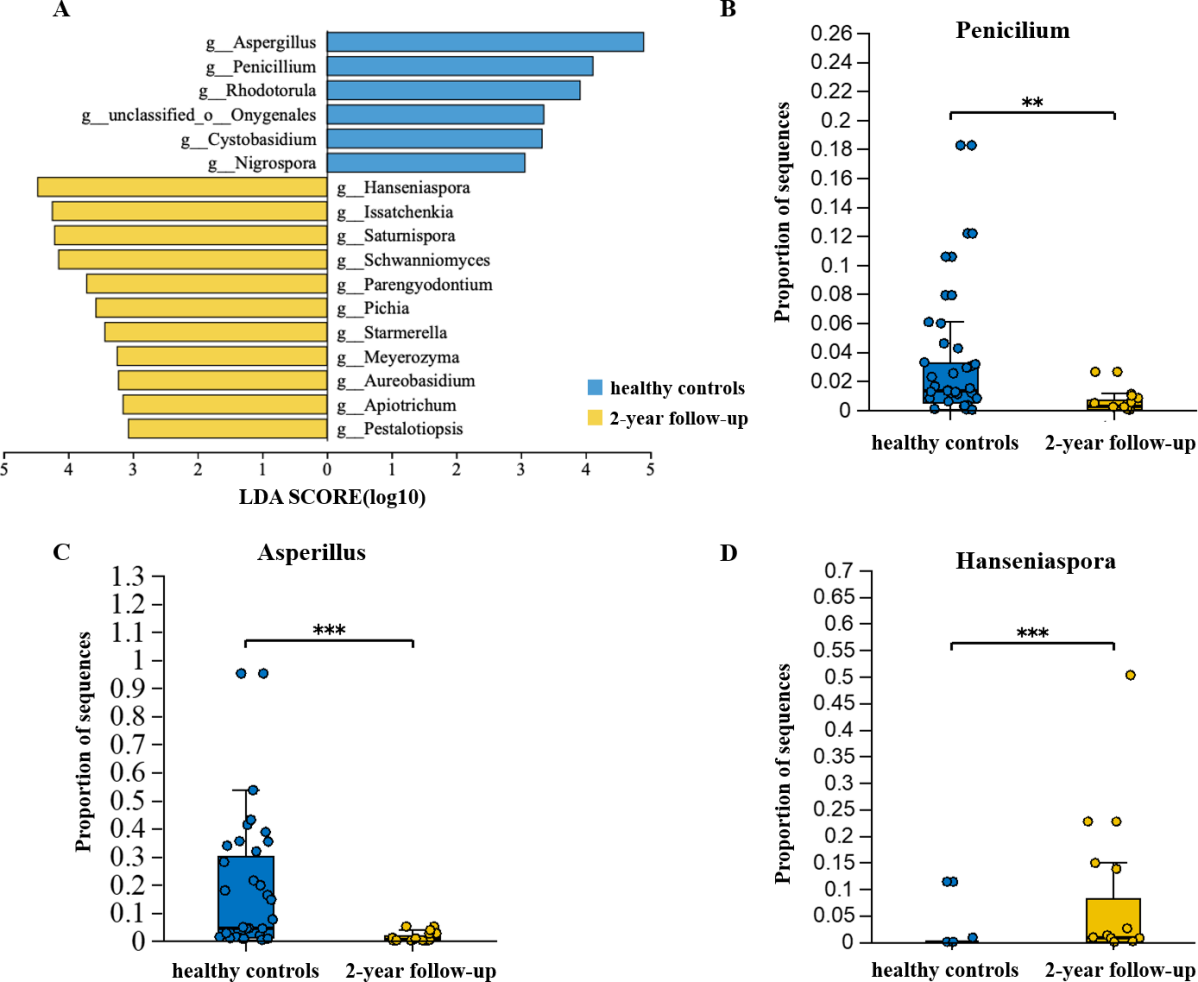
(A) Linear discrimination analysis (LDA) effect size (LEfSe) analysis was used to differentiate between patients at 2-year follow-up (yellow) and healthy controls (blue). Only taxa with an LDA threshold >3 are shown. (**B–D**) Comparative analysis of fungi genera identified by LEfSe analysis across different groups. Student’s t-test or Mann-Whitney U test was used when applicable.

Representative taxa with significant differences in abundance between the 2-year follow-up and the control group were identified using the LEfSe method. A total of 17 genera showed differential abundance between the two groups (Fig. 4A). The gut mycobiota at the 2-year follow-up exhibited higher levels of *Hanseniaspora*, *Issatchenkia*, and *Saturnispora*, while the abundances of *Aspergillus*, *Penicillium*, and *Rhodotorula* were lower (Fig. 4B through D).

**Fig 5:**
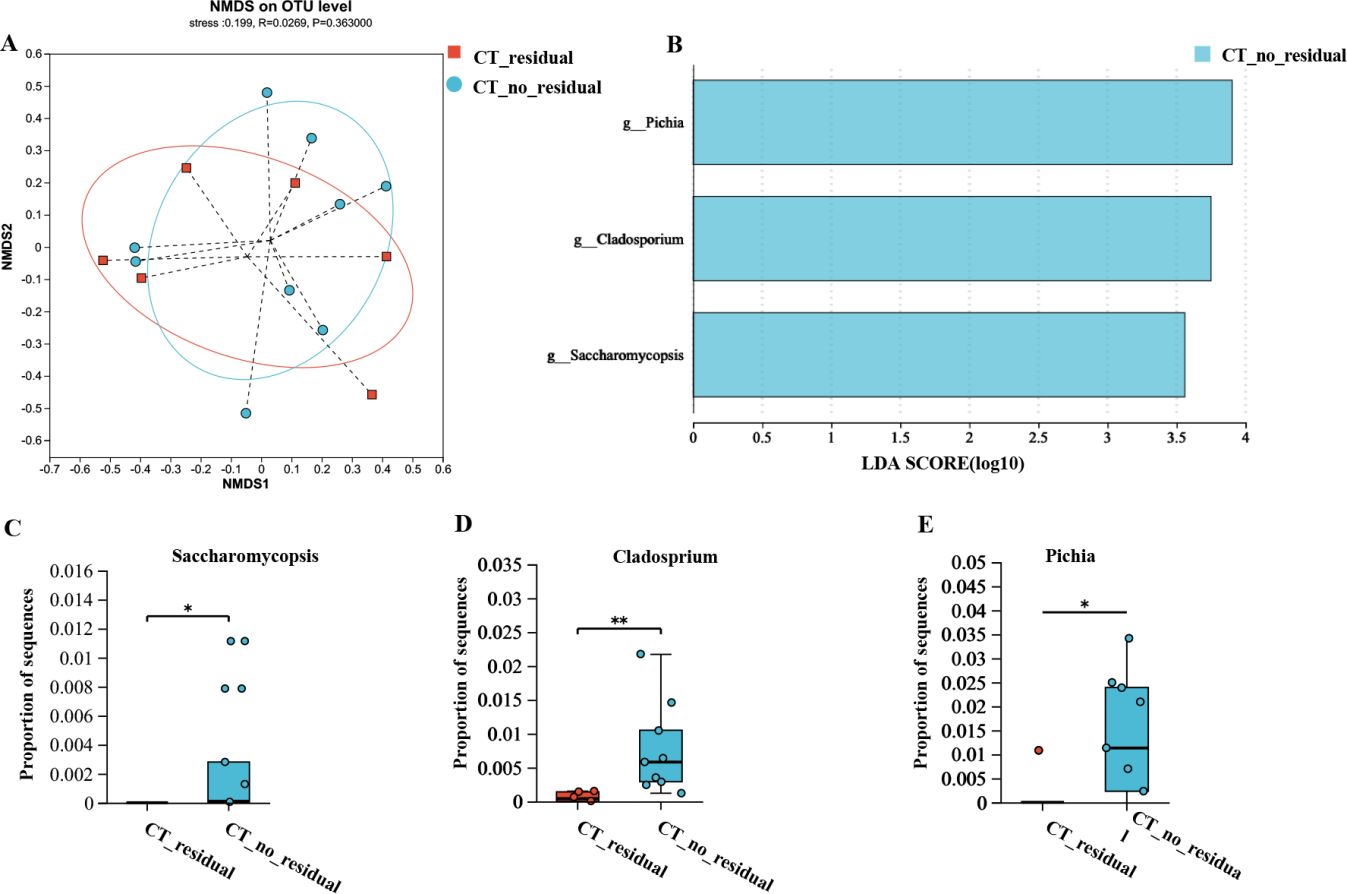
Gut mycobiota analysis in patients with CT residual capacity and those without CT residual capacity at 2-year follow-up. (**A**) NMDS ordination based on Bray-Curtis similarities showed no significant difference in mycobiota community composition between patients with CT residual capacity and those without CT residual capacity (stress = 0.199, ANOSIM, R = 0.0269, *P* = 0.363) at 2 years after discharge. (**B**) Linear discrimination analysis (LDA) effect size (LEfSe) analysis was used to differentiate between patients with or without CT residual abnormalities (blue). Only taxa with an LDA threshold >3 are shown. (**C–E**) Comparative analysis of fungi genera identified by LEfSe analysis across different groups. Student’s t-test or Mann-Whitney U test was used when applicable.

The correlations of residual radiological abnormalities with gut microbiota and mycobiota were analyzed. Fifteen patients underwent pulmonary CT examinations 2 years after discharge. They were divided into two groups based on the presence (*n* = 7) or absence (*n* = 8) of residual lesions on CT 1 year after discharge. Principal coordinate analysis based on Bray–Curtis dissimilarities revealed no significant differences in the mycobiota community compositions between patients with and without residual CT findings at the 2-year follow-up (Fig. 5A). The genera *Pichia*, *Saccharomycopsis*, and *Cladosporium* were identified as enriched in patients without residual CT findings at the 2-year follow-up (Fig. 5B through E).

**Fig 6:**
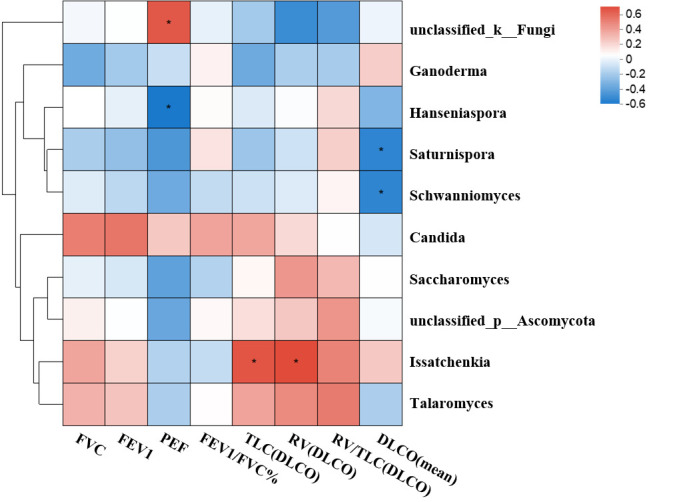
Association between fungi genera and pulmonary function test results. The Spearman correlation coefficient of the top 10 abundant fungal genera and the pulmonary function index were calculated and displayed on the heat map. Levels of significance for correlation coefficients were adjusted by Bonferroni’s correction. R in different colors to show, the right side of the legend is the color range of different R values. The degree of correlation is indicated by a color gradient from blue (negative correlation) to red (positive correlation). *P < 0.05.

We further analyzed the correlation between lung function and gut fungal composition (Fig. 6). Results showed that *Hanseniaspora* was negatively correlated with PEF. In addition, *Saturnispora* and *Schwanniomyces* were negatively correlated with the diffusion capacity of the lungs for carbon monoxide (DLCO). *Issatchenkia* was positively correlated with total lung capacity (TLC) and residual volume (RV).

## DISCUSSION

Gut microbial disturbances caused by COVID-19 have been well documented in previous studies; however, little is known about the recovery process of gut mycobiota during and after COVID-19. In our prospective, 2-year longitudinal study using ITS sequencing, we found that gut mycobiota generally recovered within 6 months post-COVID-19. In addition, alterations in gut mycobiota showed close correlations with pulmonary function.

Previous studies have shown that COVID-19 infection leads to significantly reduced alpha diversity in the gut microbiome (22, 23), with microbial richness and diversity not fully restored after 6 months of recovery (24, 25). In a prior study, Maeda et al. analyzed gut mycobiota from 40 severe COVID-19 patients, 38 mild cases, and 30 healthy individuals and reanalyzed samples from 10 severe patients approximately 6 months after discharge (15). Their results indicated that while bacterial microbiota alterations persisted post-recovery, the diversity of the mycobiota rebounded to levels comparable with those of mild cases and healthy controls. Our findings similarly suggest that fungal microbiome diversity was basically restored within 6 months. A potential reason for this relatively fast recovery of the gut mycobiota compared to bacteria could be the fungi’s ability to reproduce quickly through spore formation. These spores can withstand unfavorable conditions for extended periods and can rapidly germinate and proliferate when the environment improves. Although bacteria also have high reproductive rates, some may be more sensitive to environmental changes.

Our results showed that the genus *Candida* was overrepresented while *Aspergillus* was depleted in the acute phase, consistent with previous findings indicating an enrichment of *Candida* and a depletion of *Aspergillus* in acute COVID-19 cases (15, 16, 19). Studies on the impact of COVID-19 on the epidemiology of candidemia revealed that the incidence of candidemia nearly tripled during the COVID-19 era (2020–2023), with a marked increase in both ICU and non-ICU settings (26–30). Common risk factors contributing to this link between COVID-19 and candidemia include the use of antibiotics, corticosteroids, venous catheters, and dialysis. Furthermore, previous studies have supported that the gut is a significant source of candidemia, as COVID-19-induced mucosal barrier disruption may facilitate the translocation of *Candida* species from the gut to the bloodstream (31). Our findings also support the role of the gastrointestinal tract as a key contributor to the increase in *Candida* infections. Both *Aspergillus* and *Candida* are known to be major pathogens in COVID-19-associated invasive fungal infections (32). It is widely accepted that *Aspergillus* infections are primarily acquired through inhalation of airborne conidia (33), and our observation of depleted gut *Aspergillus* suggests that the intestine is not a significant source of *Aspergillus* infection.

Our findings indicated that pulmonary function remained well preserved in patients 2 years post-recovery. In addition, we observed that *Hanseniaspora* in the gut mycobiome was negatively correlated with peak expiratory flow (PEF), *Saturnispora* was negatively correlated with diffusion capacity of the lungs for carbon monoxide (DLCO), and *Issatchenkia* was positively correlated with residual volume (RV). LEfSe analysis further showed that these three fungal genera (*Hanseniaspora*, *Saturnispora*, and *Issatchenkia*) were significantly elevated in the 2-year recovery cohort. This suggests that gut mycobiome abnormalities may be linked to minor declines in lung function, potentially serving as a pathogenic mechanism for long COVID. *Issatchenkia orientalis* is a non-conventional yeast known for its tolerance to highly acidic conditions and ability to produce ethanol in acidic environments (34, 35). *Hanseniaspora uvarum*, *Saturnispora silvae*, and *Issatchenkia orientalis* have been detected in pit mud yeast communities used for the production of Chinese strong-flavored liquor (36). The enrichment of these yeasts in recovered COVID-19 patients may imply an acidic gut microenvironment in these individuals. In addition, residual SARS-CoV-2 has been shown to persist in patients who have recovered from mild COVID-19, with significant associations observed between viral persistence and long COVID symptoms (37). This persistent viral shedding may contribute to alterations in the gut microenvironment following SARS-CoV-2 infection.

In our previous study, we reported that 47% of patients exhibited residual radiologic lesions 1 year post-discharge, which correlated with lung volume parameters (9). At the 2-year follow-up, lung CT scans were performed on 15 patients, with 7 still showing residual lung lesions. Patients who declined to return for follow-up CT scans may have experienced better recovery, suggesting that the observed proportion of patients with residual CT abnormalities might be skewed toward a higher value. Nevertheless, these findings indicate that lung imaging recovery may be slower than anticipated. The genera *Pichia*, *Saccharomycopsis*, and *Cladosporium* were enriched in patients without residual CT findings at the 2-year mark, suggesting these fungi may have a potential beneficial effect on lung health.

*Saccharomyces* and *Pichia* were found to be positively correlated with body mass index in individuals of healthy weight compared to those who were overweight, suggesting a potential role for these taxa in nutrient acquisition (38, 39). Gut *Pichia* has also been linked to the pathophysiology of non-alcoholic steatohepatitis (NASH) through fructose-dependent endogenous alcohol and triglyceride production (40). In people with HIV, *Pichia* species showed a strong correlation with the bacterial genus *Faecalibacterium* (41). Levels of *Saccharomycetales spp.* and *Pichia* decline from middle age to later life and are inversely associated with fasting glucose, while positively associated with high-density lipoprotein cholesterol, suggesting a possible beneficial role for these fungal taxa in human metabolic health (42). Zhang et al. investigated the correlation between plasma metabolites and residual lung lesions in recovered COVID-19 patients, finding that indolelactate levels were positively associated with residual CT lesions (43). This result suggests that a higher abundance of indolelactate-producing species may lead to elevated serum levels of indolelactate, potentially contributing to ongoing inflammatory processes (44).

This study has some limitations. First, it was a single-center study with a relatively small sample size. Second, we followed only patients infected with the original strain and did not compare the effects of infections from different viral strains on gut mycobiota. Third, the associations between individual mycobiota taxons and pulmonary function also appear superficial and need more evidence linking mycobiome composition to pulmonary function in future studies. In the future, larger sample sizes and extended longitudinal studies will be essential to clarify the long-term health consequences attributable to COVID-19, as well as the different influences of variable variants.

In conclusion, this study reveals the longitudinal shifts in gut mycobiota over 2 years post-discharge in COVID-19 patients. Significant correlations were observed between pulmonary function and the gut mycobiota, suggesting a potential detrimental effect on lung function, offering potential insights into the pathogenesis of post-acute COVID-19 syndromes.

## Data Availability

The raw sequence data reported in this paper have been deposited in the Genome Sequence Archive (Genomics, Proteomics & Bioinformatics 2021) in the National Genomics Data Center (Nucleic Acids Res 2022), the China National Center for Bioinformation/Beijing Institute of Genomics, the Chinese Academy of Sciences (GSA: CRA020039), and are publicly accessible at https://ngdc.cncb.ac.cn/gsa.

## SUPPLEMENTAL MATERIAL

Supplemental material spectrum.03007-24-s0001.docx Table S1; Fig. S1 and S2.

ASM does not own the copyrights to Supplemental Material that may be linked to, or accessed through, an article. The authors have granted ASM a non-exclusive, world-wide license to publish the Supplemental Material files. Please contact the corresponding author directly for reuse.
