# Supplementary material for: Longitudinal alterations of gut mycobiota during 2 years after COVID-19 and its correlation with pulmonary sequela
Source: Microbiol Spectr. 2025 May 23;13(7):e03007-24. doi: 10.1128/spectrum.03007-24 (PMC12210925; doi:10.1128/spectrum.03007-24)
Supplement: Supplemental material — Table S1; Fig. S1 and S2. [file spectrum.03007-24-s0001.docx]

**Supplementary materials**

| **Supplementary Tabe S1. Comparison of pulmonary funtion test among different follow-up timepoints** | | | |
| --- | --- | --- | --- |
|  | 6 months after discharge (n=33) | 12 months after discharge (n=29) | 2 years after discharge (n=18) |
| DLCO < 80%, n (%) | 10 (30.3%) | 2 (7.1%)* | 1 (5.5%)* |
| TLC < 80%, n (%) | 2 (6.1%) | 4 (14.3%) | 1 (5.5%) |
| FEV1/FVC < 0.70, n (%) | 3 (9.1%) | 3 (10.7%) | 0 (0%) |
| FVC | 96 (88, 105) | 96.5 (85.75, 102.75) | 95 (90.25, 105.5) |
| FEV1 | 96 (87, 107) | 98 (86.25, 105.25) | 97 (86.25, 102.75) |
| PEF | 83 (72, 101) | 85.5 (71, 100) | 88 (79.5, 98.75) |
| FEV1/FVC% | 80.7 (75.7, 87.9) | 82.7 (78.8, 85.325) | 102.5 (97.25, 104.75) |
| TLC(DLCO) | 96 (91, 105) | 96 (87.75, 104.5) | 97.5 (90.25, 104.5) |
| RV(DLCO) | 117 (103, 130) | 119 (104.25, 137.25) | 109 (99.25, 122.75) |
| DLCOmean | 88 (77, 95) | 112.5 (104.5, 121)** | 95 (89.5, 109.75)* |
| The occurrence data are shown as no. (%) unless otherwise indicated. Values indicate no. of positive results/total no. of patients with available assay results. Paired Student’s T-Test or Mann-Whitney U test (for quantitative data) and McNemar test (for category data) were used to compare the results of two follow-up timepoints. Abbreviations: PFTs, pulmonary function tests; FVC, forced vital capacity; FEV1, forced expiratory volume in the first 1 second of expiration; PEF, peak expiratory flow; DLCO, diffusing capacity of the lung for carbon monoxide; TLC, total lung capacity; RV, residual volume. Pulmonary function tests were expressed as percent of the predicted value. * indicated P<0.05 when compared to the results of 6-month follow-up; ** indicated P<0.01 when compared to the results of 6-month follow-up. | | | |

**Supplementary Figure S1** A. Chao 1 α-diversity in patients with severe illness was significantly lower during the acute phase of hospitalization than during convalescence and compared to healthy controls. B. Chao 1 α-diversity in hospitalized patients with mild illness showed no significant difference between the acute and recovery phases compared to healthy controls. C. α-diversity of Shannon α-diversity in patients with severe illness was significantly lower during the acute phase of hospitalization than during convalescence and compared to healthy controls. D. Shannon α-diversity in hospitalized patients with mild illness showed no significant difference between the acute and recovery phases compared to healthy controls. For the same sample at different time points, paired Student’s T-Test or Mann-Whitney U test was used depending on whether the data was normally distributed. For comparing each time point with the healthy control group, an unmatched two-sample Student’s T-Test or Mann-Whitney U test was used depending on whether the data was normally distributed. *p < 0.05, **p < 0.01, ***p < 0.001

**Supplementary Figure S2.** Beta diversity difference analysis was performed using intra-group distance measures for Bray-Curtis distances of OTUs. This analysis revealed significant differences in community structure among groups by calculating the intra-group sample distance index. The points in the box plot represent the pairwise sample distances within each group. Kruskal-Wallis H test was used to compare significant difference among groups.
